# Differential DNA methylation in discrete developmental stages of the parasitic nematode *Trichinella spiralis*

**DOI:** 10.1186/gb-2012-13-10-r100

**Published:** 2012-10-17

**Authors:** Fei Gao, Xiaolei Liu, Xiu-Ping Wu, Xue-Lin Wang, Desheng Gong, Hanlin Lu, Yudong Xia, Yanxia Song, Junwen Wang, Jing Du, Siyang Liu, Xu Han, Yizhi Tang, Huanming Yang, Qi Jin, Xiuqing Zhang, Mingyuan Liu

**Affiliations:** 1 Key Lab for Zoonosis Research, Ministry of Education, Institute of Zoonosis, Jilin University; Zoonosis Research Centre of State Key Lab for Molecular Virology and Genetic Engineering, Chinese Academy of Medical Sciences, 5333 Xi An Road, Changchun, 130062, China; 2 Science and Technology Department, BGI-Shenzhen, Beishan Industrial Zone, Yantian District, Shenzhen 518083, China

## Abstract

**Background:**

DNA methylation plays an essential role in regulating gene expression under a variety of conditions and it has therefore been hypothesized to underlie the transitions between life cycle stages in parasitic nematodes. So far, however, 5'-cytosine methylation has not been detected during any developmental stage of the nematode *Caenorhabditis elegans*. Given the new availability of high-resolution methylation detection methods, an investigation of life cycle methylation in a parasitic nematode can now be carried out.

**Results:**

Here, using MethylC-seq, we present the first study to confirm the existence of DNA methylation in the parasitic nematode *Trichinella spiralis*, and we characterize the methylomes of the three life-cycle stages of this food-borne infectious human pathogen. We observe a drastic increase in DNA methylation during the transition from the new born to mature stage, and we further identify parasitism-related genes that show changes in DNA methylation status between life cycle stages.

**Conclusions:**

Our data contribute to the understanding of the developmental changes that occur in an important human parasite, and raises the possibility that targeting DNA methylation processes may be a useful strategy in developing therapeutics to impede infection. In addition, our conclusion that DNA methylation is a mechanism for life cycle transition in *T. spiralis *prompts the question of whether this may also be the case in any other metazoans. Finally, our work constitutes the first report, to our knowledge, of DNA methylation in a nematode, prompting a re-evaluation of phyla in which this epigenetic mark was thought to be absent.

## Background

Developmental regulation of gene expression plays a crucial role in the transitions between significantly differentiated life-history stages, such as is the case in parasitic nematodes; however, the underlying mechanisms of this gene regulation are poorly understood. Although DNA methylation has been established in other organisms as an important method for altering chromatin structure and regulating the expression of genes, its contribution to nematode development has not been adequately assessed given that so far no 5' cytosine methylation has been identified in any stage of *Caenorhabditis elegans *[1]. Most vertebrate cell types have approximately 60 to 90% of the CpG dinucleotides modified to 5-methylcytosine (5mC) [2], whereas invertebrate genomes vary extensively in the extent of DNA methylation, and some genomes have undetectable levels of methylation [3]. Recently, technological progress has enabled high-resolution detection of 5mC, opening the way for more detailed examination of the role of DNA methylation in a greater variety of eukaryotic genomes [4].

Parasitic nematodes are a good example of the biological importance of developmental regulation of genes, including the principal agent of human trichinellosis, *Trichinella spiralis*. This food-borne agent infects a wide variety of vertebrate hosts through their ingestion of meat containing encysted muscle larvae (ML). ML are released by the host's gastric juices, after which they grow substantially and mature into sexually active adults (Ad) in the host's intestines. New-born larvae (NBL) are released from mature females and then disseminate through the bloodstream, invade skeletal muscles, and encyst in a collagen capsule to form a new generation of ML [5]. The host muscle cells proliferate as they are transformed into 'nurse cells' for the parasite [6]. The major clinical symptoms of trichinellosis (myopathy) derive from inflammation directed against the encysted ML. Thus, successful nematode development entails a series of physically and functionally distinct stages that require accurate recognition of specific biological cues. In this way, the life cycle of parasitic nemotodes is distinct from that of free-living nematodes, such as *Caenorhabditis elegans*, that live in a more homogeneous environment.

Stage-specific expression has been observed for genes in *Trichinella *spp. [7]. Differential expression was especially obvious for genes encoding the excretory-secretory (E-S) proteins released from the larvae. For example, a gene encoding a 43-kDa glycoprotein is expressed in precapsular and postcapsular muscle larvae, but not in adults [8]. E-S proteins may therefore contribute to capsule formation [9]. Stage-specific gene expression may also assist parasitic evasion or forestalling of immune reactions that would inhibit continued transmission. Thus, how stage-specific transcriptional regulation is accomplished in these organisms might prove useful for understanding and preventing infection.

Recent innovations in high-throughput sequencing have enabled researchers to infer methylation patterns at single-base resolution [10]. MethylC-seq enables methylation analyses with unprecedented precision, and the recently released draft genome sequence of *T. spiralis *[11] provided us the means to evaluate the methylome of its three distinct stages. Our work here describes the first comprehensive study that confirms the existence of DNA methylation in *T. spiralis *and characterizes the differential methylomes of the organism during these life stages. We further identified sets of genes whose DNA methylation status varied between the developmental stages. Our data shed light on the developmental biology of an important food-borne zoonosis, and our approach opens the way for future assessment of methylation as a mechanism of developmental regulation in this and other metazoans that undergo similar life cycle transitions.

## Results

### The presence of DNA methylation in the *T. spiralis *genome

**Figure 1:**
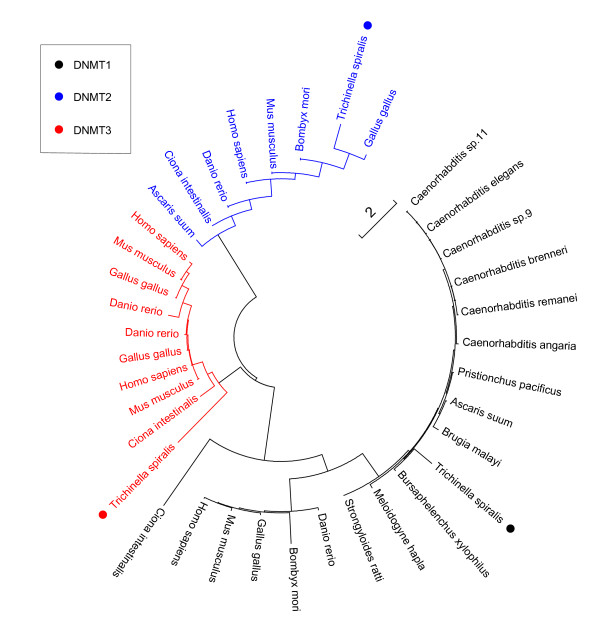
**Phylogenetic tree of dnmt proteins**. Multiple sequence alignment was performed by ClusterW, then ClusterW with the neighbor-joining method based on JTT+ G (Jones-Taylor-Thornton and Gamma Distribution) model was applied to reconstruct the phylogenetic tree. The species with best hits to *T. spiralis *dnmts were used as representatives that span the phylum and were analyzed in this study.

To understand whether *T. spiralis *possesses the ability to methylate DNA, we conducted reciprocal Blast searches to identify genes that might be related to known DNA (cytosine-5)-methyltransferases. Our data revealed the existence of several relevant orthologous genes annotated in the draft *T. spiralis *genome [11] (Table S1 in Additional data file 1). We found that EFV54759.1 and EFV58204.1 were homologous to dnmt3 *de novo *methyltransferases and to the maintenance methyltransferase dnmt1, respectively, in species that are known to have DNA methylation, such as human and mouse. Of additional interest, *T. spiralis *appeared to be the only nematode, compared to 11 other nemotodes, that possessed *de novo *methylation machinery (dnmt3). The other nematodes only contained orthologs to maintenance methyltransferase dnmt1, including *Caenorhabditis elegans*. We also identified an ortholog to dnmt2 (EFV60295.1), but it was more similar to a previously identified tRNA methylase [12-14], which suggests the potential existence of RNA methylation in *T. spiralis*. We used the sequences of these dnmt-like proteins to reconstruct a phylogenetic tree (Figure 1). This analysis indicated that *T. spiralis *dnmt3 was not a close relative of orthologs in its host mammals, suggesting that *T. spiralis *dnmt3 did not originate from its host through horizontal gene transfer.

We performed PCR with reverse transcription (RT-PCR) and found that *T. spiralis *dnmt2 and dnmt3 genes were differentially expressed among three life stages, but that dnmt1 expression remained at about the same level (Figure S1a in Additional data file 2). Correspondingly, enzymatic data using nuclear protein extracts also showed differential catalytic activity of the *T. spiralis *dnmts (Figure S1b in Additional data file 2). We also carried out ultra-performance liquid chromatography-tandem mass spectrometry (UPLC-MS/MS), which further confirmed the existence of DNA methylation in *T. spiralis*, showing that the total amount of DNA methylation in the Ad stage was significantly higher than in the NBL stage (Figure S2 in Additional data file 2) [15].

Given these results, we assessed the genome-wide DNA methylation profiles in the three life stages of *T. spiralis *(Ad, ML, and NBL) using MethylC-Seq. We generated 61.65, 23.52 and 55.77 million raw reads, respectively. We aligned the reads to the *T. spiralis *reference sequence [16] and mapped approximately 96.36% of the reads to Ad, 91.30% to ML and 99.27% to NBL, yielding 2.91, 1.05 and 2.71 Gb of DNA sequence to Ad, ML, and NBL, respectively. The average read depth was 21.36, 10.80 and 26.21 per strand, respectively. On average, over 81.6% of each strand of the 64 Mb *T. spiralis *reference sequence was covered by at least one sequence read in each of the three stages. Because of the potential for the occurrence of non-conversion and thymidine-cytosine sequencing errors, we estimated the false-positive rate as the percentage of cytosines sequenced at cytosine reference positions in the Lambda genome, which are normally unmethylated (Materials and methods). We then applied the error rates for each stage (0.0060, 0.0064 and 0.0025 for Ad, ML, and NBL, respectively) to correct mC identification according to a method described by Lister *et al*. [10] that is based on a binomial test and false discovery rate constraints. Corrected estimates resulted in approximately 0.31 million and 0.24 million mCs in the Ad and ML genomes (comprising 1.59% and 1.22% of their sequenced cytosines, respectively). In contrast, methylation was nearly undetectable in NBL (0.002 million; 0.01%; Table S2 in Additional data file 2). We validated the results using two different methods: (1) bisulfite-PCR (BSP), cloning, and conventional sequencing by the Sanger method; and (2) methylated DNA immunoprecipitation (MeDIP) combined with quantitative PCR (QPCR). For BSP, we assessed six randomly selected genomic regions that varied in their estimated amount of methylation, and obtained strong agreement between the two experimental results (*P*-value < 0.05 using double *t*-test; Figure S3 in Additional data file 2; Table S3 in Additional data file 1) [15]. For MeDIP with QPCR, we assessed three randomly selected genomic regions and confirmed the existence of DNA methylation in all three regions (Figure S4 in Additional data file 2).

### Characterization of overall methylation patterns in the three life stages

**Figure 2:**
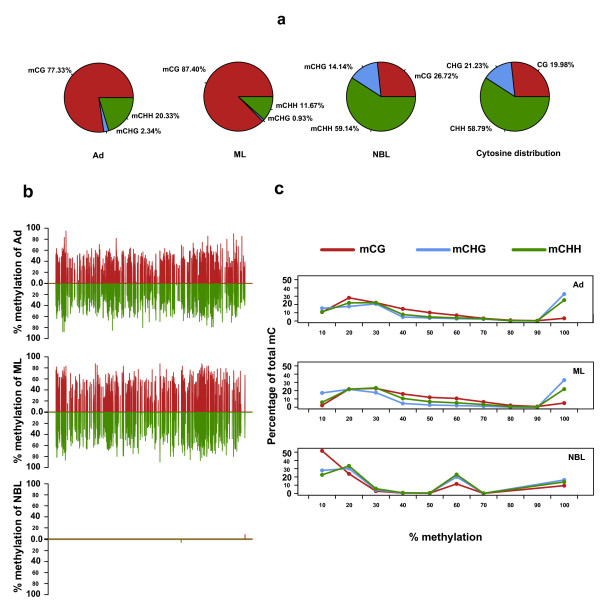
**DNA methylation patterns and chromosomal distribution of three life stages of *T. spiralis***. **(a) **The fraction of mCs identified in each sequence context in the three life stages in comparison with the fraction of all Cs in each sequence context in the genome. **(b) **Distribution of MRs identified on the two DNA strands (Watson and Crick) throughout the whole genome. The value refers to the average percentage of methylation of the MRs, as shown on the y-axis. **(c) **Distribution of mCs (y-axis) across the percentage methylation levels (x-axis).

We further characterized the global patterns of DNA methylation in the genomes of the different *T. spiralis *stages. The highest amount of detected mCs (82.25% and 89.06%) in Ad and ML were located in CG regions, indicating a dominant role of CpG methylation in these stages. Due to the very low level of DNA methylation in NBL, the distribution of CG and non-CG methylation was very similar to the background (Figure 2a). The average methylation level of specific cytosine residues could be estimated from the fraction of methylated sequence reads at that site. Here we found that the average methylation level of specific cytosine residues was estimated from the fraction of methylated sequence reads at that site (Figure 2c).

Since the mCs in the *T. spiralis *genome are relatively sparse compared to vertebrate genomes, we identified methylation regions (MRs) of the genome using relatively dense mCs (Materials and methods). Different CG and non-CG methylation might be subject to distinct forms of genetic control; therefore, MR identification was performed independently for CG and non-CG contexts. Across the genome, we observed an increase in CG methylation as the parasites matured from the NBL to the ML stage and, to a lesser extent, in the transition from ML to Ad. In addition, CG methylation levels fluctuated drastically across the genome, indicating a mosaic methylation pattern where relatively dense methylated domains are interspersed with regions that are not methylated (Figure 2b). Such a pattern has been observed in previous studies on other invertebrates [3]. In contrast, we identified only a small number of non-CG MRs (Table S4 in Additional data file 1).

**Figure 3:**
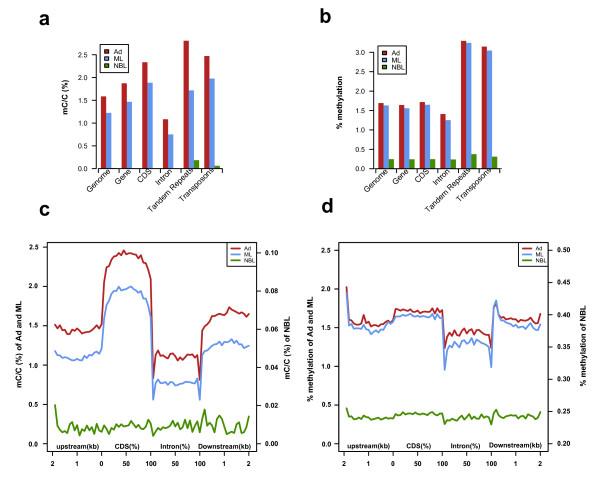
**Average methylation levels of different genomic regions of the three life stages of *T. spiralis***. **(a, b) **Average density of methylation levels (a) and percentage of methylation levels (b) (y-axis; Materials and methods) at different functional regions (x-axis). **(c, d) **Average density of methylation levels (c) or percentage of methylation levels (d) (y-axis) of intervals around genic regions (x-axis). Two-kilobase regions upstream and downstream of each gene were divided into 100-bp (bp) intervals. Each coding sequence or intron was divided into 20 intervals (5% per interval).

In all types of genomic elements, we saw a methylation increase from NBL to ML and from ML to Ad as well as the global pattern. We then examined patterns of methylation in distinct genomic elements, including genes, tandem repeats, and transposable elements. Genes were methylated more frequently than the genome average (Figure 3a, b). Within genes, the coding sequences were more methylated than the flanking DNA or promoter regions, while introns were the least methylated (Figure 3c, d). Notably, repeat elements, including tandem repeats and transposable elements, exhibited much higher DNA methylation than the genome average (Figure 3a, b). Previous studies have indicated that the methylation level of transposons across different phylogenetic units may vary. It has been reported that transposable elements are highly methylated in mammals, plants and zebrafish (*Danio rerio*), and moderately methylated in *Ciona intestinalis*, but are usually unmethylated in the honey bee *Apis mellifera *and silkworm *Bombyx mori *[17,18]. In *T. spiralis*, we observed higher methylation on transposons relative to the immediate flanking regions as well (Figure S5 in Additional file 2), which is similar to what is seen in *Ciona intestinalis *[17].

### The relationship between stage-dependant methylation and gene expression

We evaluated differential gene expression among the three life stages using Illumina high-throughput RNA-seq technology. Most of the raw reads (numbering 28,662,704, 26,128,346 and 28,962,820, respectively, for the Ad, ML and NBL stages) could be uniquely mapped to previously annotated genes (62.26%, 64.38%, and 64.34%). We detected 12,675, 12,683 and 12,909 annotated genes out of the total 16,379 with at least one unique read. The majority of these genes (11,636) were expressed in all three life stages, and we saw 234 Ad-stage specific, 183 ML-specific and 445 NBL-specific genes. Of note, we also detected stage-dependent expression of methyltransferases that were concordant with prior RT-PCR results (Figure S2 in Additional data file 2). Finally, among genes that were expressed in more than one stage, we identified differential expression in 1,752 pair-wise comparisons (Table S5 in Additional data file 1).

**Figure 4:**
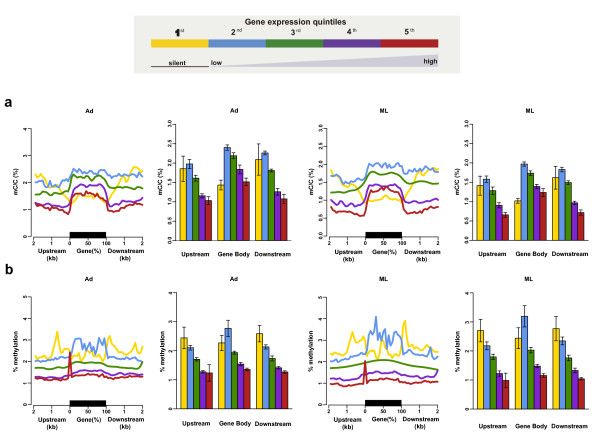
**Relationship between DNA methylation and expression levels of genes in Ad and ML stages of *T. spiralis***. **(a, b) **Average density (a) or percentage of methylation levels (b) within genes that were classified based on expression levels. The first class includes silent genes with no sequencing read detected, and the second to fifth classes cover expressed genes from the lowest 25% to highest 25%. In the curve graph, 2-kb regions upstream and downstream of each gene are divided into 100-bp intervals, and each gene was divided into 20 intervals (5% per interval). In the histogram graph, overall average (± standard error) methylation levels for genes are indicated.

We characterized the changes in DNA methylation among the three distinct life stage methylomes and the relationship between methylation and differential gene expression. For this, we divided expressed genes with at least one sequencing read into quartiles of expression levels, and examined the expressed genes together with another category composed of genes exhibiting no expression. We found that DNA methylation levels of gene upstream regions had a negative correlation with gene expression levels, and non-expressed genes in particular had different patterns of DNA methylation as the methylation levels in their upstream regulatory regions were higher than in the coding sequences (Figure 4a, b). Based on this, it is likely that methylated promoters induce silencing in *T. spiralis*, akin to the widely accepted role for hypermethylation of promoters as a means of repressing gene expression in plants and mammals [19,20]. In contrast, the gene-body methylation levels in our analysis showed a bell-shaped, rather than monotonic, relationship with gene expression levels. Generally in the gene body, the non-expressed and most highly expressed genes had the lowest DNA methylation levels, whereas the mid-level expressed genes had the highest percentage of DNA methylation (Figure 4a-c). A bell-shaped relationship between gene-body methylation and expression levels has been observed previously in plants (*Arabidopsis thaliana *and *Oryza sativa*), invertebrates (*Ciona intestinalis *and *Nematostella vectensis*), and humans as well [4,21,22], indicating conservation of the role of methylation across phylogenetically diverse species.

### Biological implications of stage-dependant methylation in *T. spiralis*

**Figure 5:**
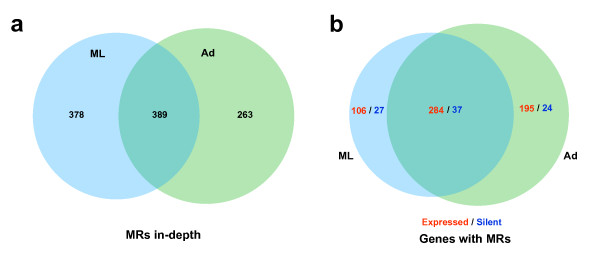
**Analysis of highly enriched MRs and annotation of the genes containing MRs**. **(a) **Venn diagram of shared and stage-specific MRs in different sequence contexts of Ad and ML stages. **(b) **Venn diagram of shared and disparate genes containing MRs in Ad and ML stages, expressed (red) and silent (blue) genes are separated.

We examined the correspondence of methylation status to divergent gene expression in different stages. Due to the overall low level of genome methylation in general, we limited this analysis to MRs exhibiting high levels of methylation where we had at least 5× depth of coverage. Using this criteria, we found a total of 652 ML and 767 Ad MRs enriched for methylation in CG regions, but MRs in non-CG regions were rare. In contrast, we found no MRs in the NBL stage. As shown in Figure 5a, 389 MRs were shared between Ad and ML. These MRs were located in 486 and 551 genes in the ML and Ad stages, respectively, with the majority located in gene-body regions (Table S4 in Additional data file 1).

**Figure 6:**
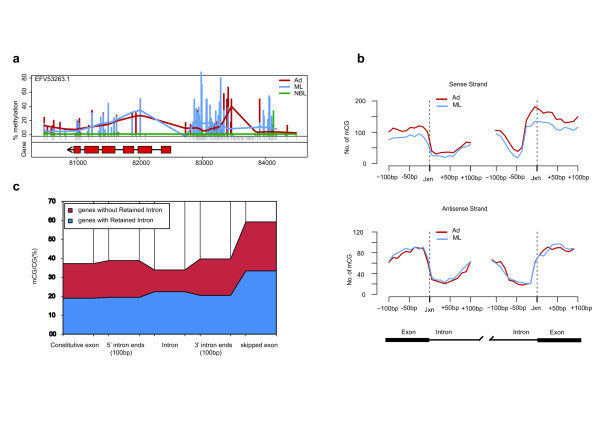
**Stage-dependant DNA methylation in relation to gene repression and alternative splicing**. **(a) **Graphical representation of differential methylation profiles in the NBL-specific DNase II gene encoding protein EFV53263.1. Ad (red line) and ML (blue line) show hypermethylation in their gene upstream regions. **(b) **Distribution of methylation on both sides of donor and acceptor sites in 20-nucleotide sliding windows of 200 nucleotides total length centered at the splice junctions (jxn). The exon-intron boundaries are shown in vertical dashed lines. **(c) **Graphical representation of methylation densities in constitutive and skipped exons, introns and 100-nucleotide 5', 3' intronic sites. The relative widths of the shaded regions correspond to methylation frequencies.

We carried out a Gene Ontology analysis to functionally characterize those genes with CG MRs in Ad and ML using GOstat [23]. Enrichment of GO terms defined by a significant false discovery rate-corrected *P*-value (≤0.01) in the 'molecular function' category was indicated in 'DNA integration', 'DNA metabolic process' and so on. In the 'biological process' category, 'nucleic acid metabolic process' and 'endopeptidase activity' and so on were enriched. Of note, we found that many genes were shared among different molecular pathways and constituted a central focus of study in parasitic nematodes (Table S6 in Additional data file 1). Given this, we explored the potential for DNA methylation regulating genes that are related to parasitic activities. For example, the protein EFV53263.1 is encoded by a DNase II gene in the 'DNA metabolic process' category that is expressed in a stage-specific manner and important for *T. spiralis *parasitism [24], and we found that this gene was uniquely transcribed in the NBL stage, whereas it was not expressed and had hypermethylated promoter regions in the Ad and ML stages. Considering the globally monotonic and negative correlation between promoter methylation and gene expression, this finding strongly suggested that promoter methylation plays a role in the regulation of stage-specific expression of certain DNase II genes in *T. spiralis *(Figure 6a).

In addition to a role for promoter methylation in expression, recent studies have suggested that gene-body methylation is involved in alternative splicing regulation. Our RNA-seq results indicated that many *T. spiralis *genes were alternatively spliced (Figure S6 in Additional data file 2), and in relation to this, we saw a steep change in the methylation frequency across splice junctions on both sense and antisense strands in the MR genes (Figure 6b). Moreover, there were considerable methylation differences between skipped and constitutive exons or between retained and spliced introns (Figure 6c). Particularly prominent was also the 'infiltration of methylation' in intronic sequences of neighboring splice junctions (Figure 6b, c). These results are in agreement with previous studies [25-27]. Taken together, our data suggest that DNA methylation status has a relationship with the donor/acceptor sequence context around the splice junctions, indicating the potential influence of DNA methylation on alternative splicing of MR genes.

## Discussion

The status of DNA methylation in nematode genomes has been unclear. Research on *Caenorhabditis elegans*, which reportedly lacked mC in age-synchronous senescent populations, indicated it had negligible influence [1]. With regard to *Caenorhabditis elegans*, our computational searches here indicate that it has dnmt1, but not dnmt2 [12] or dnmt3. As dnmt3 is essential for *de novo *methylation [28], the lack of dnmt3 in *Caenorhabditis elegans *might explain the absence of *de novo *methylation in this nemotode. In contrast, we identified three methyltransferase genes in the *T. spiralis *genome that were orthologs to known dnmts in vertebrates, including dnmt1, dnmt2 and dnmt3. Intriguingly, of 11 species of nematodes tested, *T. spiralis *appeared to be the only nematode possessing *de novo *methylation machinery (dnmt3). Moreover, we found that expression levels of dnmt2 and dnmt3 increased during development from larvae to adulthood, but that dnmt1 did not. We note that our analysis indicated dnmt2 was more similar to a tRNA methylase instead of a DNA methyltransferase [12-14], which might indicate that RNA methylation also plays a role in *T. spiralis *development, and is therefore worth more detailed analysis in the future.

We further carried out the first comprehensive, high-resolution analysis of methylation in *T. spiralis*, to assess the intriguing possibility, given the presence of *de novo *DNA methyltransferase orthologs, that epigenetic control might help govern the development of its distinct life history stages via temporally regulated gene expression. Methylome sequencing revealed a mosaic methylation pattern in *T. spiralis*, typical of other invertebrates [29,30]. DNA methylation increased drastically during maturation from NBL to ML, and adults exhibited the highest observed DNA methylation level. This finding contrasts with a trend seen in some other species where methylation patterns remain stable throughout the life cycle [29]. For instance, in the sea urchin, which also has distinct life stages, methylated and non-methylated regions in its genome retain the same overall methylation composition throughout all tested stages [31]. The relative overall constancy of methylation patterns is also a feature of vertebrate genomes. However, the extent of DNA methylation may reflect changes in both intrinsic and environmental exposure [32]. For instance, studies in humans indicated that total genomic 5-methylcytosine has been found to typically decrease during aging [33,34], in concordance with declining Dnmt1 activity with age [35]. Parasites such as *T. spiralis *certainly undergo more drastic lifespan changes in response to environmental cues, that is, metamorphosis critical to their survival and reproduction. Our findings here provide evidence to indicate that these DNA methylation changes might play an important role in regulating such transformations in *T. spiralis*.

Previous studies have indicated that methylation may be an evolutionarily ancient means of transcriptional control as it is maintained in phylogenetically diverse lineages. In both plants and vertebrates, the notion that methylation in promoters primarily represses genes by impeding transcriptional initiation has been widely accepted [19,20], whereas intermediate levels of expression have been associated with genes experiencing the greatest extent of methylation in the gene body, indicating a bell-shaped relationship [4,21,22,36]. However, in the fungus *Neurospora crassa *[37] and the silkworm *Bombyx mori *[18], transcription initiation is unaffected. Thus, DNA methylation shows remarkable diversity in its extent and function across eukaryotic evolution. Here, our results indicate that the presence of promoter methylation correlates with reduced gene expression levels. Promoter hypermethylation may regulate a portion of stage-specific genes by repressing their transcription initiation in non-expressed stages, as exemplified by a NBL-specific DNase II gene (Figure 6a). Our assessment of gene-body methylation, which had a bell-shaped relationship between methylation and gene expression, indicates there was no overt relationship between expression and methylation levels. We did, however, see evidence for a relationship between methylation within the gene-body and alternative splicing of these genes in *T. spiralis*, indicating these regulatory mechanisms of gene and protein activity are an area of interest for future study as well; and the presence of a tRNA methylase ortholog makes further study of RNA regulation in general in the organism of interest.

In relation to the notion that complex regulatory machinery of DNA methylation has been developed in *T. spiralis *with species-specific characteristics, probably in response to environmental cues, we found that many of the MR genes are enriched in pathways that are functionally important for parasitic nematodes. Such genes modulate the interaction between the parasite and its host so as to protect the parasite against host immune responses. There was also enrichment in pathways that are important to parasitic activity, including previously reported catalytically active E-S proteins. Of note, hydrolases are among the most abundant proteins secreted by parasites and facilitate host tissue invasion [38]. Also important to *T. spiralis *is the conversion of muscle cells to nurse cells, and DNA-binding proteins [39], which are often affected by methylation changes, are believed to interfere with host cell signaling in ways that promote this conversion. Additionally, many such proteins are encoded by large, developmentally regulated gene families and assume different isoforms, which is also relevant to our findings that MRs were primarily distributed in gene bodies rather than promoter regions and the DNA methylation status was related to the donor/acceptor sequence context around their splice junctions.

## Conclusions

We describe the first comprehensive study confirming the existence of DNA methylation in three life stages of *T. spiralis*. Our data also provide support for DNA methylation being associated with the regulation of genes that are closely related to the parasitism of the organism. In this context, in *T. spiralis *and in other organisms that experience discrete and highly specialized development forms, further consideration should be given to mechanisms where DNA methylation is involved in suppression of spurious transcriptional initiation of infrequently transcribed genes, promotes transcriptional termination, or mediates alternative splicing, as has been shown for other model organism systems.

## Materials and methods

### Collection of *T. spiralis *muscle larvae, adults and new-born larvae

Infective *T. spiralis *ML were obtained from infected mice at 35 days post-infection by digestion of minced skeletal muscle in 1% pepsin and 1% HCl for 45 minutes at 42°C with agitation, as previously described [40]. Seventy male 6-week-old Wistar rats were then orally inoculated with a dose of 8,000 infective ML. Adult worms (Ad1) were obtained from the intestine of ten rats at 30 h post-infection. The remaining 60 rats were sacrificed at 6 days post-infection, and the adult worms (Ad6) were recovered and incubated in Iscove's Modified Dulbecco's Medium (IMDM) in 75-cm^2 ^cell culture plates at 37°C. Newborn larvae were harvested every 6 h. All experiments were performed in accordance with the Guide for the Care and Use of Laboratory Animals published by the National Institutes of Health (publication no. 85-23, revised 1996). The protocol was approved by the Ethical Committee of the Institute of Zoonosis, Jilin University, China (reference number 20080106).

### Enzymatic activity analysis of Dnmts

To test the dnmt enzymatic activity of *T. spiralis*, 11 μg of nuclear extracts for each assay were incubated in 37°C for 2 h using a EpiQuik™ DNMT Activity/Inhibition Assay Ultra Kit (Epigentek, Farmingdale, NY, USA) according to the manufacturer's instructions.

### BlastP searches and phylogenetic analysis of Dnmts

Reciprocal BlastP comparisons were first performed to identify dnmt orthologs. Significant hits were defined as those satisfying the following criteria: E-value < 10^-5 ^and the aligned segments covering at least 30% of the sequence length of the hit. For phylogenetic analysis, multiple sequence alignment was performed by ClusterW [41]. The ClusterW with the neighbor-joining method [42] based on JTT+ G (Jones-Taylor-Thornton and Gamma Distribution) model was applied to reconstruct the phylogenetic tree.

### UPLC-MS/MS analysis of global DNA methylation

UPLC-MS/MS analysis was performed according to a previously published method [43]. Genomic DNA (0.2 μg) extracted from Ad and NBL was digested with 1U DNase I, 2U Alkaline Phoaphatase, Calf Intestinal (CIP) and 0.005U snake venom phosphodiesterase I at 37°C for 24 h. A microcon centrifugal filter device with a 3,000 D cutoff membrane was used to remove protein from the digested DNA samples by centrifuging at 12,000 rpm for 60 minutes. The mobile phase, consisting of 5.0% methanol and 95% water (plus 0.1% formic acid), was used for UPLC separation of the nucleotides at a flow rate of 0.25 ml/minute. Enzymatically digested DNA samples (10 μl each) were injected for UPLC-MS/MS analysis and each run took 10 minutes. Mass spectrometry conditions were as follows: ionizationmode, ESI-positive; capillary voltage, 3,500 V; nitrogen drying gas temperature, 300°C; drying gas flow, 9 L/min; nebulizer, 40 psi. For MS/MS analysis of nucleotides, the fragmentor voltage was 90 V, collision energy was performed at 5 eV and scan time was 100 ms. Multiple-reaction monitoring (MRM) mode was used for the UPLC-MS/MS analysis by monitoring transition pairs of m/z 242.1/126.0 corresponding to 5mdC. The isotope labeled internal standard (5mdC-d3) was used to quantify genomic DNA methylation level, whose m/z was 245.4/129.0.

### MethylC-seq library construction and sequencing

Prior to library construction, 5 μg of genomic DNA spiked with 25 ng unmethylated Lambda DNA (Promega, Madison, WI, USA) was fragmented using a Covarias sonication system to a mean size of approximately 200 bp. After fragmentation, libraries were constructed according to the Illumina Pair-End protocol with some modifications. Briefly, purified randomly fragmented DNA was treated with a mix of T4 DNA polymerase, Klenow fragment and T4 polynucleotide kinase to repair, blunt and phosphorylate the ends. The blunt DNA fragments were subsequently 3' adenylated using Klenow fragment (3'-5' exo-), followed by ligation to adaptors synthesized with 5'-methylcytosine instead of cytosine using T4 DNA ligase. After each step, DNA was purified using the QIAquick PCR purification kit (Qiagen, Shanghai, China). Next, a ZYMO EZ DNA Methylation-Gold Kit™ (ZYMO Research, Irvine, CA, USA) was employed to convert unmethylated cytosine into uracil, according to the manufacturer's instructions, and 220 to 250 bp converted products were size selected. Finally, PCR was carried out in a final reaction volume of 50 μl, consisting of 20 μl of size-selected fractions, 4 μl of 2.5 mM dNTP, 5 μl of 10× buffer, 0.5 μl of JumpStart™ Taq DNA Polymerase, 2 μl of PCR primers and 18.5 μl water. The thermal cycling program was 94°C for 1 minute, 10 cycles of 94°C for 10 s, 62°C for 30 s, 72°C for 30 s, and then a 5-minute incubation at 72°C, before holding the products at 12°C. The PCR products were purified using the QIAquick gel extraction kit (Qiagen). Before analysis with Illumina Hiseq2000, the purified products were analyzed by the Bioanalyser analysis system (Agilent, Santa Clara, CA, USA) and quantified by real time PCR. Raw sequencing data were processed using the Illumina base-calling pipeline (Illumina Pipeline v1.3.1). The sodium bisulfite non-conversion rate was calculated as the percentage of cytosines sequenced at cytosine reference positions in the Lambda genome.

### RNA-sequencing and real-time PCR validation

Total RNA was extracted using the Invitrogen TRIzol^®^ Reagent and then treated with RNase-free DNase I (Ambion, Guangzhou, China) for 30 minutes. The integrity of total RNA was checked using an Agilent 2100 Bioanalyser. cDNA libraries were prepared according to the manufacturer's instructions (Illumina). The poly(A)-containing mRNA molecules were purified using Oligo(dT) Beads (Illumina) from 20 μg of total RNA from each sample. Tris-HCl (10 mM) was used to elute the mRNA from the magnetic beads. To avoid priming bias when synthesizing cDNA, mRNA was fragmented before the cDNA synthesis. Fragmentation was performed using divalent cations at an elevated temperature. The cleaved mRNA fragments were converted into double-stranded cDNA using SuperScript II, RNaseH and DNA Pol I, primed by random primers. The resulting cDNA was purified using the QIAquick PCR Purification Kit (Qiagen). Then, cDNA was subjected to end repair and phosphorylation using T4 DNA polymerase, Klenow DNA polymerase and T4 Polynucleotide Kinase (PNK). Subsequent purifications were performed using the QIAquick PCR Purification Kit (Qiagen). These repaired cDNA fragments were 3'-adenylated using Klenow Exo- (Illumina) and purified using the MinElute PCR Purification Kit (Qiagen), producing cDNA fragments with a single 'A' base overhang at the 3' end for subsequent ligation to the adapters. Illumina PE adapters were ligated to the ends of these 3**'**-adenylated cDNA fragments and then purified using the MinElute PCR Purification Kit (Qiagen). To select a size range of templates for downstream enrichment, the products of the ligation reaction were purified on a 2% TAE- Certified Low-Range Ultra Agarose (Bio-Rad, Hercules, CA, USA). cDNA fragments (200 ± 20 bp) were excised from the gel and extracted using the QIAquick Gel Extraction Kit (Qiagen). Fifteen rounds of PCR amplification were performed to enrich the adapter-modified cDNA library using primers complementary to the ends of the adapters (PCR Primer PE 1.0 and PCR Primer PE 2.0; Illumina). The 200 ± 20 bp PCR products were purified using QIAquick Gel Extraction Kit (Qiagen), using the MinElute spin columns (Qiagen). Finally, after detection on an Agilent Technologies 2100 Bioanalyser using the Agilent DNA 1000 chip kit and quantification on a StepOne plus qPCR (ABI, Woodlands, Singapore), the cDNA library products were sequenced using the Illumina Genome Analyser. Real-time PCR validation was conducted using the Maxima^®^ SYBR^®^ Green qPCR Master Mix kit (Fermentas, Beijing, China), according to the manufacturer's instructions, in an ABI Prism 7500 Sequence Detection System machine (Applied Biosystems Inc., CA, USA). All real-time RT-PCR data were normalized to the NBL stage (see Additional data file 1 for primer information).

### Sequence alignment of MethylC-seq

The reads generated by Illumina sequencing were aligned onto the *T. spiralis *reference genome [11]. The Lambda genome was also included in the reference sequence as an extra chromosome so that reads originating from the unmethylated control DNA could be aligned. Because DNA methylation has strand specificity, the plus strand and the minus strand of the *T. spiralis *genome were separated to form alignment target sequences. To do this, each cytosine in the genome was converted to a thymine, termed the T-genome, which represented the plus strand. Meanwhile, each guanine in genome sequences was converted to adenosine, termed the A-genome, which represented the minus strand. Additionally, the original forms of the reads were also transformed to deal with the bisulfite treatment nucleotide conversion in the alignment process. First, the observed cytosines on the forward read of each read pair were replaced *in silico *by thymines, and secondly, the observed guanines on the reverse read of each read pair were replaced *in silico *by adenosines. We then mapped the 'alignment form' reads to the 'alignment form' target sequence using SOAPaligner with default parameters [16]. Every hit of a single placement with a minimum number of mismatches and a clear strand assignment was defined as an unambiguous alignment, and each alignment was used for mC ascertainment.

### Gene annotation and functional analysis

For gene annotation, the BLAST algorithm was applied to further annotate the genes defined in the available *T. spiralis *genome annotation because the current annotation is incomplete. All the predicted protein sequences of *T. spiralis *genes were aligned using BLAST with known annotated protein sequences from three databases, including SWISS-Prot, TrEMBL and InterPro. A cutoff E-value < 1e-05 was applied for annotation, and a best alignment term for each query protein sequence was selected if more than query sequence was aligned based on this cutoff E-value from BLAST.

For function analysis, GO analysis was performed based on the annotated genes by GOstat software [23]; 8,286 genes with annotation out of 16,380 genes were used as background, and 287 and 242 genes with annotation out of the 540 and 454 genes containing MRs in Ad and ML, respectively, were used as input genes. Fisher's exact test was performed and the *P*-values generated for each GO category were adjusted according to the Benjamini and Hochberg correction method.

### Identification of methylcytosines and methylation regions and determination of methylation level

For mC identification, we transformed each aligned read and the two strands of the *T. spiralis *genome back to their original forms to build an alignment. In the unique part of the genome, cytosines that were covered by cytosines from reads on the same strand or guanines from those on the opposite strand (hereafter, referred to as ascertainment bases) were called as potential methylated sites. To account for the inefficient conversion of the bisulfite treatment and for sequencing errors, a correction method based on binomial tests and false discovery rate constraints [10] was applied to the data to build a high-quality methylome for each of the three stages. The probability *p *in the binomial distribution B(n, p) was estimated from the number of cytosine bases sequenced in reference cytosine positions in the unmethylated Lambda genome (referred to as the error rate: non-conversion plus sequencing error frequency). The bisulfite conversion rates for all samples were over 99%, and the error rates were as follows: Ad, 0.0060; ML, 0.0064; NBL, 0.0025. Then, the mCs from the binomial distribution analysis were selected for determination of MRs, which were defined as being under a threshold of more than five continuous mCs (either CpG or non-CpGs in at least one strand) and having a distance between adjacent mCs of less than the median value (18 bp) of all distance values. Stage-specific MRs were defined as containing more than five continuous mCs and no overlap between two samples.

Percentage methylation was computed as the fraction of reads number of 'C' in the total reads number of 'C' and 'T' for each covered CpG site, and herein average percentage methylation of all cytosine residues for any genomic region covered was computed as the fraction of reads number of 'C' in the total reads number of 'C' and 'T' for each genomic region. Density methylation (mC/C) was determined as the number of mCs divided by total number of C sites in any genomic region.

### Validation of DNA methylation

Two strategies were applied to validate the methylation status of randomly selected genomic regions. BSP combined with cloning Sanger sequencing. BSP primers were designed by the online MethPrimer software (Additional data file 1). Genomic DNA (500 ng) was converted using the ZYMO EZ DNA Methylation-Gold Kit™ according to the manufacturer's instructions. PCR amplification was carried out with a thermal cycling program of 94°C for 1 minute, 30 cycles of 94°C for 10 s, 58°C for 30 s, 72°C for 30 s, and then 5 minutes at 72°C. The products were then held at 12°C. Following amplification, PCR products were gel selected and purified using the QIAquick gel extraction kit (Qiagen), and the purified PCR products were subcloned. The colonies from each region were sequenced on a 3730 genetic analyxer (Applied Biosystems) to determine the methylated cytosine levels.

MeDIP followed by QPCR (see Additional data file 1 for primer information) was performed on 300 to 400 ng of original genomic DNA for each sample, which was randomly sheared to an average length of 200 to 500 bp by sonication. A MeDIP assay was then performed using the Magnetic Methylated DNA Immunoprecipitation Kit (Diagenode, Liege, Belgium) according to the instructions. The immunoprecipitated products and 10% amount of original input DNA were purified with ZYMO DNA Clean & Concentrator-5 kit (ZYMO) in parallel. The purified DNA was analyzed by QPCR on an ABI StepOne Plus Real Time PCR System (Applied Biosystems Inc.) using Eva Green (Biotium, Shanghai, China). The relative methylation levels of particular genomic loci among samples were compared by measuring the amount of immunoprecipitated DNA after normalization to the 10% of input DNA: %(MeDNA-IP/Total input) = 2^[Ct(10%input) - 3.32 - Ct(MeDNA-IP)] × 100%.

### Data availability

Sequence data and processed data are available under the Gene Expression Omnibus accession GSE39328. UPLC-MS/MS and BS-PCR data have been deposited in GigaDB, the GigaScience database, with the unique identifier doi:10.5524/100043 [15].

## Abbreviations

Ad: adult; BSP: bisulfite-PCR; E-S: excretory-secretory; Gb: gigabase; GO: Gene Ontology; mC: methylcytosine; MeDIP: methylated DNA immunoprecipitation; ML: muscle larvae; MR: methylation region; NBL: new-born larvae; QPCR: quantitative PCR; UPLC-MS/MS: ultra-performance liquid chromatography-tandem mass spectrometry.

## Authors' contributions

FG, ML, QJ, XZ and HY conceived the project. FG designed experiments and interpreted data. XL, XPW, XLW, YS, JW, JD, XH, and YT performed experiments. FG, DG, HL, YX and SL conducted bioinformatic analysis. FG prepared and wrote the manuscript. All authors have read and approved the manuscript for publication.

## Supplementary Material

Additional file 1 **Supplemental Tables S1 to S6 and corresponding captions**. Click here for file

Additional file 2 **Information on all primer sequences, and Supplemental Figures S1 to S6 and corresponding legends**. Click here for file

## References

[B1] Simpson VJ Johnson TE Hammen RF Caenorhabds elegans DNA does not contain 5-methylcytosine at any time during development or aging. Nucleic Acids Res 1986 13 9 10.1093/nar/14.16.6711 PMC311675 3748820

[B2] Bird A DNA methylation patterns and epigenetic memory. Genes Dev 2002 13 6 21 10.1101/gad.947102 11782440

[B3] Tweedie S Charlton J Clark V Bird A Methylation of genomes and genes at the invertebrate-vertebrate boundary. Mol Cell Biol 1997 13 1469 1475 9032274 10.1128/mcb.17.3.1469 PMC231872

[B4] Zemach A McDaniel IE Silva P Zilberman D Genome-wide evolutionary analysis of eukaryotic DNA methylation. Science 2010 13 916 919 10.1126/science.1186366 20395474

[B5] Janssen CS Tetley L Kennedy MW Developmental activation of infective Trichinella spiralis larvae. Parasitology 1998 13 363 371 10.1017/S003118209800314X 9820858

[B6] Gottstein B Pozio E Nockler K Epidemiology, diagnosis, treatment, and control of trichinellosis. Clin Microbiol Rev 2009 13 127 145 10.1128/CMR.00026-08 19136437 PMC2620635

[B7] Mitreva M Jasmer DP Appleton J Martin J Dante M Wylie T Clifton SW Waterston RH McCarter JP Gene discovery in the adenophorean nematode Trichinella spiralis: an analysis of transcription from three life cycle stages. Mol Biochem Parasitol 2004 13 277 291 10.1016/j.molbiopara.2004.05.015 15383298

[B8] Wu Z Nagano I Nakada T Takahashi Y Expression of excretory and secretory protein genes of Trichinella at muscle stage differs before and after cyst formation. Parasitol Int 2002 13 155 161 10.1016/S1383-5769(02)00003-X 12113753

[B9] Nagano I Wu Z Takahashi Y Functional genes and proteins of Trichinella spp. Parasitol Res 2009 13 197 207 10.1007/s00436-008-1248-1 18987885

[B10] Lister R Pelizzola M Dowen RH Hawkins RD Hon G Tonti-Filippini J Nery JR Lee L Ye Z Ngo QM Edsall L Antosiewicz-Bourget J Stewart R Ruotti V Millar AH Thomson JA Ren B Ecker JR Human DNA methylomes at base resolution show widespread epigenomic differences. Nature 2009 13 315 322 10.1038/nature08514 19829295 PMC2857523

[B11] Mitreva M Jasmer DP Zarlenga DS Wang Z Abubucker S Martin J Taylor CM Yin Y Fulton L Minx P Yang SP Warren WC Fulton RS Bhonagiri V Zhang X Hallsworth-Pepin K Clifton SW McCarter JP Appleton J Mardis ER Wilson RK The draft genome of the parasitic nematode Trichinella spiralis. Nat Genet 2011 13 228 235 10.1038/ng.769 21336279 PMC3057868

[B12] Kunert N Marhold J Stanke J Stach D Lyko F A Dnmt2-like protein mediates DNA methylation in Drosophila. Development 2003 13 5083 5090 10.1242/dev.00716 12944428

[B13] Goll MG Kirpekar F Maggert KA Yoder JA Hsieh CL Zhang X Golic KG Jacobsen SE Bestor TH Methylation of tRNAAsp by the DNA methyltransferase homolog Dnmt2. Science 2006 13 395 398 10.1126/science.1120976 16424344

[B14] Jurkowski TP Meusburger M Phalke S Helm M Nellen W Reuter G Jeltsch A Human DNMT2 methylates tRNA(Asp) molecules using a DNA methyltransferase-like catalytic mechanism. RNA 2008 13 1663 1670 10.1261/rna.970408 18567810 PMC2491481

[B15] Gao F Wang J Ji G Bisulfite-PCR combined with cloning Sanger sequencing data for validating DNA methylation level in Trichinella spiralis. GigaScience 2012 http://dx.doi.org/10.5524/100043

[B16] Li R Li Y Kristiansen K Wang J SOAP: short oligonucleotide alignment program. Bioinformatics 2008 13 713 714 10.1093/bioinformatics/btn025 18227114

[B17] Feng S Cokus SJ Zhang X Chen PY Bostick M Goll MG Hetzel J Jain J Strauss SH Halpern ME Ukomadu C Sadler KC Pradhan S Pellegrini M Jacobsen SE Conservation and divergence of methylation patterning in plants and animals. Proc Natl Acad Sci USA 2010 13 8689 8694 10.1073/pnas.1002720107 20395551 PMC2889301

[B18] Xiang H Zhu J Chen Q Dai F Li X Li M Zhang H Zhang G Li D Dong Y Zhao L Lin Y Cheng D Yu J Sun J Zhou X Ma K He Y Zhao Y Guo S Ye M Guo G Li Y Li R Zhang X Ma L Kristiansen K Guo Q Jiang J Beck S Single base-resolution methylome of the silkworm reveals a sparse epigenomic map. Nat Biotechnol 2010 13 516 520 10.1038/nbt.1626 20436463

[B19] Zhang X The epigenetic landscape of plants. Science 2008 13 489 492 10.1126/science.1153996 18436779

[B20] Weber M Hellmann I Stadler MB Ramos L Paabo S Rebhan M Schubeler D Distribution, silencing potential and evolutionary impact of promoter DNA methylation in the human genome. Nat Genet 2007 13 457 466 10.1038/ng1990 17334365

[B21] Zilberman D Gehring M Tran RK Ballinger T Henikoff S Genome-wide analysis of Arabidopsis thaliana DNA methylation uncovers an interdependence between methylation and transcription. Nat Genet 2007 13 61 69 10.1038/ng1929 17128275

[B22] Jjingo D Conley AB Yi SV Lunyak VV Jordan IK On the presence and role of human gene-body DNA methylation. Oncotarget 2012 13 462 474 22577155 10.18632/oncotarget.497 PMC3380580

[B23] Beissbarth T Speed TP GOstat: find statistically overrepresented Gene Ontologies within a group of genes. Bioinformatics 2004 13 1464 1465 10.1093/bioinformatics/bth088 14962934

[B24] Liu MF Wu XP Wang XL Yu YL Wang WF Chen QJ Boireau P Liu MY The functions of Deoxyribonuclease II in immunity and development. DNA Cell Biol 2008 13 223 228 10.1089/dna.2007.0691 18419230

[B25] Malousi A Kouidou S DNA hypermethylation of alternatively spliced and repeat sequences in humans. Mol Genet Genomics 2012 13 631 642 10.1007/s00438-012-0703-y 22740315 PMC3407362

[B26] Laurent L Wong E Li G Huynh T Tsirigos A Ong CT Low HM Kin Sung KW Rigoutsos I Loring J Wei CL Dynamic changes in the human methylome during differentiation. Genome Res 2010 13 320 331 10.1101/gr.101907.109 20133333 PMC2840979

[B27] Lyko F Foret S Kucharski R Wolf S Falckenhayn C Maleszka R The honey bee epigenomes: differential methylation of brain DNA in queens and workers. PLoS Biol 2010 13 e1000506 10.1371/journal.pbio.1000506 21072239 PMC2970541

[B28] Goll MG Bestor TH Eukaryotic cytosine methyltransferases. Annu Rev Biochem 2005 13 481 514 10.1146/annurev.biochem.74.010904.153721 15952895

[B29] Bird AP Taggart MH Variable patterns of total DNA and rDNA methylation in animals. Nucleic Acids Res 1980 13 1485 1497 10.1093/nar/8.7.1485 6253937 PMC324011

[B30] Wang Y Jorda M Jones PL Maleszka R Ling X Robertson HM Mizzen CA Peinado MA Robinson GE Functional CpG methylation system in a social insect. Science 2006 13 645 647 10.1126/science.1135213 17068262

[B31] Bird AP Taggart MH Smith BA Methylated and unmethylated DNA compartments in the sea urchin genome. Cell 1979 13 889 901 10.1016/0092-8674(79)90329-5 487434

[B32] Jaenisch R Bird A Epigenetic regulation of gene expression: how the genome integrates intrinsic and environmental signals. Nat Genet 2003 13 Suppl 245 254 12610534 10.1038/ng1089

[B33] Wilson VL Smith RA Ma S Cutler RG Genomic 5-methyldeoxycytidine decreases with age. J Biol Chem 1987 13 9948 9951 3611071

[B34] Bollati V Schwartz J Wright R Litonjua A Tarantini L Suh H Sparrow D Vokonas P Baccarelli A Decline in genomic DNA methylation through aging in a cohort of elderly subjects. Mech Ageing Dev 2009 13 234 239 10.1016/j.mad.2008.12.003 19150625 PMC2956267

[B35] Casillas MA Jr Lopatina N Andrews LG Tollefsbol TO Transcriptional control of the DNA methyltransferases is altered in aging and neoplastically-transformed human fibroblasts. Mol Cell Biochem 2003 13 33 43 10.1023/A:1025548623524 14577574

[B36] Sarda S Zeng J Hunt BG Yi SV The Evolution of Invertebrate Gene Body Methylation. Mol Biol Evol 2012 10.1093/molbev/mss062 22328716

[B37] Rountree MR Selker EU DNA methylation inhibits elongation but not initiation of transcription in Neurospora crassa. Genes Dev 1997 13 2383 2395 10.1101/gad.11.18.2383 9308966 PMC316521

[B38] Dzik JM Molecules released by helminth parasites involved in host colonization. Acta Biochim Pol 2006 13 33 64 16410836

[B39] Mak CH Ko RC DNA-binding activity in the excretory-secretory products of Trichinella pseudospiralis (Nematoda: Trichinelloidea). Parasitology 2001 13 301 308 11578094 10.1017/s0031182001008459

[B40] Liu MY Wang XL Fu BQ Li CY Wu XP Le Rhun D Chen QJ Boireau P Identification of stage-specifically expressed genes of Trichinella spiralis by suppression subtractive hybridization. Parasitology 2007 13 1443 1455 10.1017/S0031182007002855 17475093

[B41] Larkin MA Blackshields G Brown NP Chenna R McGettigan PA McWilliam H Valentin F Wallace IM Wilm A Lopez R Thompson JD Gibson TJ Higgins DG Clustal W and Clustal X version 2.0. Bioinformatics 2007 13 2947 2948 10.1093/bioinformatics/btm404 17846036

[B42] Saitou N Nei M The neighbor-joining method: a new method for reconstructing phylogenetic trees. Mol Biol Evol 1987 13 406 425 3447015 10.1093/oxfordjournals.molbev.a040454

[B43] Wang X Suo Y Yin R Shen H Wang H Ultra-performance liquid chromatography/tandem mass spectrometry for accurate quantification of global DNA methylation in human sperms. J Chromatogr B Analyt Technol Biomed Life Sci 2011 13 1647 1652 10.1016/j.jchromb.2011.04.002 21536504

