# Supplementary material for: Differential DNA methylation in discrete developmental stages of the parasitic nematode Trichinella spiralis
Source: Genome Biol. 2012 Oct 17;13(10):R100. doi: 10.1186/gb-2012-13-10-r100 (PMC4053732; doi:10.1186/gb-2012-13-10-r100)
Supplement: Additional file 2 — Information on all primer sequences, and Supplemental Figures S1 to S6 and corresponding legends. [file gb-2012-13-10-r100-S2.PDF]

Differential methylation regulates global gene expression in discrete developmental stages of the parasitic nematode *Trichinella spiralis*

Fei Gao, Xiaolei Liu, Xiu-Ping Wu, Xue-Lin Wang, Desheng Gong, Hanlin Lu, Yudong Xia, Yanxia Song, Junwen Wang, Jing Du, Siyang Liu, Xu Han, Yizhi Tang, Huanming Yang, Qi Jin, Xiuqing Zhang, Mingyuan Liu

Supplemental Data

1, Primers used in RT-PCR for *T. spiralis* dnmts:

Gene_ID	Protein_ID	Forward primer	Reverse primer
Tsp_05801	EFV60295.1	TGAGGAACGAACGAACACCACGA	TCGTACGCACCAACGGGAACG
Tsp_00737	EFV58204.1	GGCGGGTTCCACGCGAACAT	TCGACCGAACGGACTGGGCT
Tsp_09280	EFV54759.1	GGCCGAATCGACGAGGCGTT	AACAACGCCGGGCCACCAAA

2, Primers used in BSP:

Contig ID	Forward Primer	Reverse Primer	size
ABIR02004848.1:1028:1249	GTTTTATTAAATTTTTTAGTTAATGTA	CAACCTTATAATCCATAACCC	221bp
ABIR02000010.1:8512:8687	TTTTTTTGTTTAATTATTTGTGATT	CTAAATAAACCCCAAACTCCTTTCT	175bp
ABIR02000918.1:205130:205353	ATAATAATAATATTGATAATATGGTGGTGA	TTCTTTCAACAAAAACAAAAAAAAC	224bp
ABIR02001908.1:224428:224642	TTGGTTATGGTTATGTTTGGTAGAG	CAAAAAATTCCTTAAATTACCTTCC	215bp
ABIR02000831.1:184335:184584	GGATTGGTGTAGTGTAAGTTAT	AACAACAACAACAACAACAAATAAA	250bp
ABIR02000831.1:185504:185794	TTGTTTTTGGGTTTTGGGTAA	TTATACAAATTCAACTACCTATTAAC	291bp

3, Primers used in MeDIP-QPCR:

Related Gene	Contig ID		Forward Primer	Reverse Primer	size
EFV53250.1	ABIR02001432.1	35118-35276	GATGCAGAGCCGAGTGCGG	ACTGCGCTATCCTCGGCTCCA	159bp
EFV58106.1	ABIR02000756.1	166791-166925	GGACAGCCGATAAAGCGCC	TGCCCGTCATTGAAGGTGGG	135bp
EFV62279.1	ABIR02000064.1	108698-108830	GATGCAGAGCCGAGTGCGG	ACTGCGCTATCCTCGGCTCCA	133bp

Figure S1

a

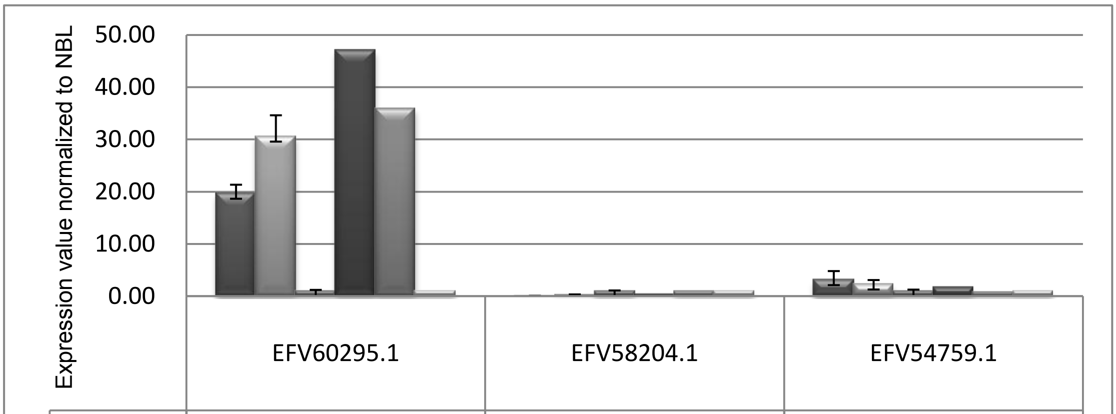

	EFV60295.1	EFV58204.1	EFV54759.1
Ad_PCR	19.65	0.09	3.14
ML_PCR	30.59	0.31	2.30
NBL_PCR	1.00	1.00	1.00
Ad_SEQ	47.02	0.37	1.68
ML_SEQ	35.93	0.95	0.80
NBL_SEQ	1.00	1.00	1.00

b

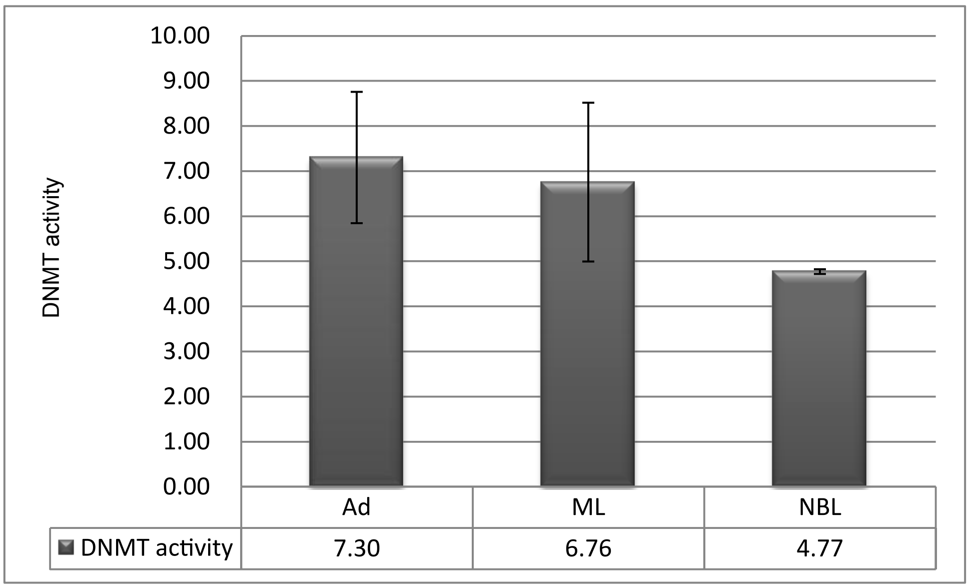

	Ad	ML	NBL
DNMT activity	7.30	6.76	4.77

Figure S1. a, results of RT-PCR and RNA-seq for expression of *T. spiralis* dnmt genes. Expression levels of all genes are normalized to data of NBL, which are indicated in y axis. Triplicates of each RT-PCR reaction were carried out, and ±standard deviation are indicated; b, results of catalytic activity analysis of *T. spiralis* dnmts. Triplicates of DNMT activity experiment were carried out, and ±standard deviation are indicated. DNMT activity (OD/h/mg) = $\frac{\text{(Sample OD} - \text{Blank OD)}}{\text{Protein amount (ug)} \times \text{hour}}$.

Figure S2

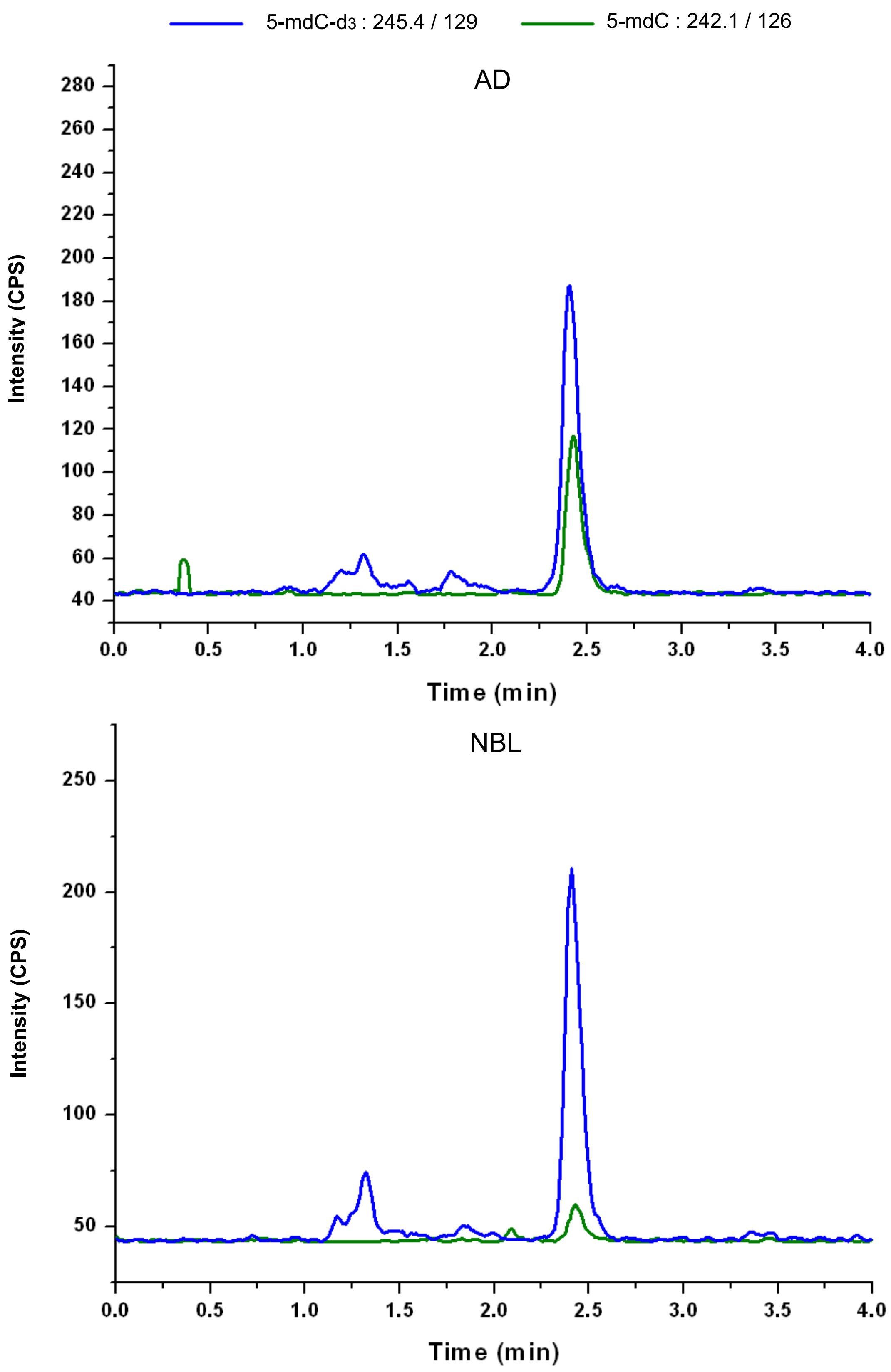

Figure S2. UPLC–MS/MS chromatograms of DNA hydrolysate from Ad and NBL. 5mdC and 5mdC-d3 were detected by monitoring m/z 242.1/126 and 245.4/129.0, respectively.

Figure S3

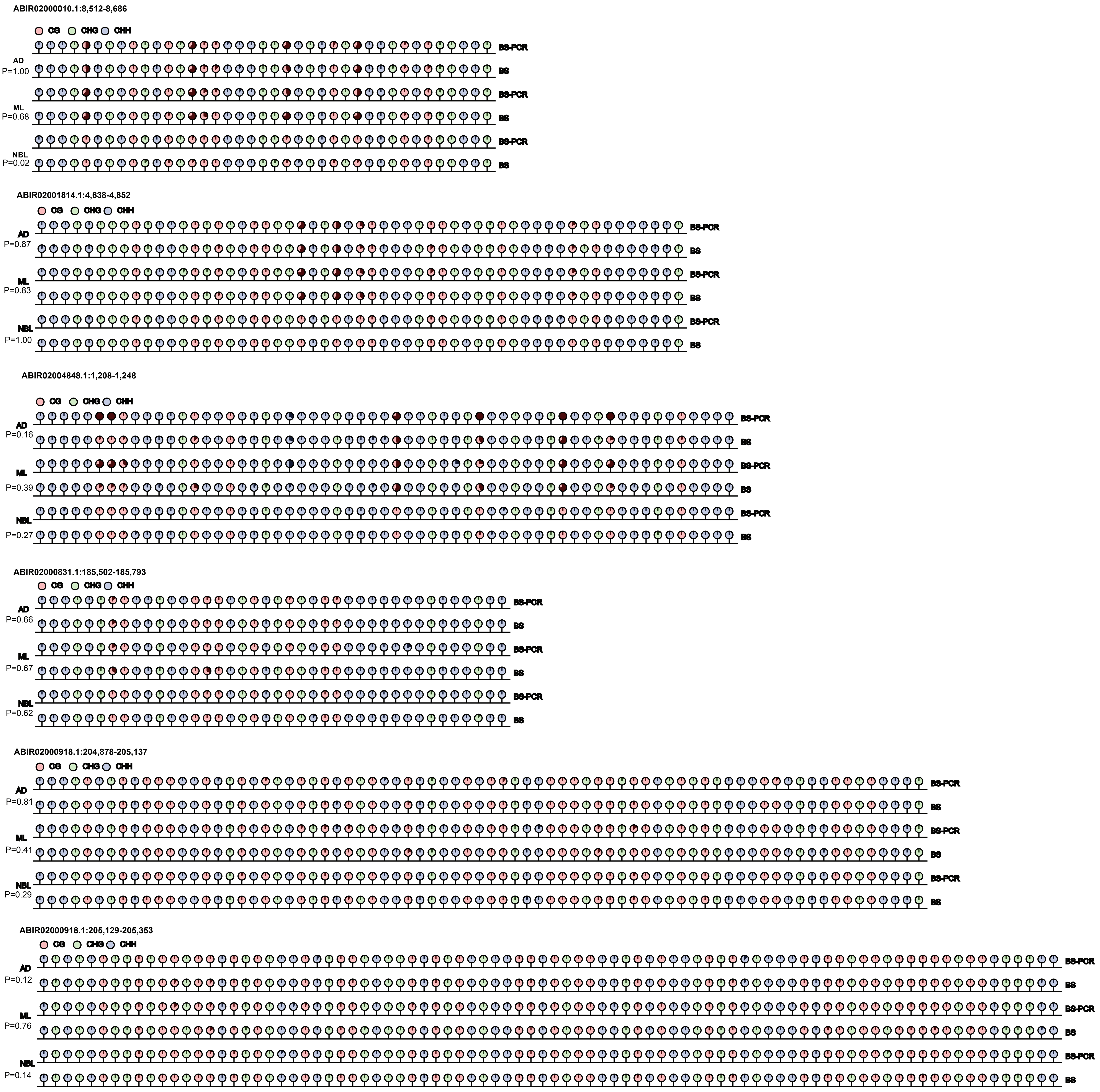

Figure S3. Results of BSP validation on six randomly selected genomic regions in three life stages. DNA methylation sequence context is displayed according to the key and the percentage methylation at each position is represented by the fill of each circle (see Table S3 in Additional data file 1 for values). P-values of double t-test are indicated for each comparison.

Figure S4

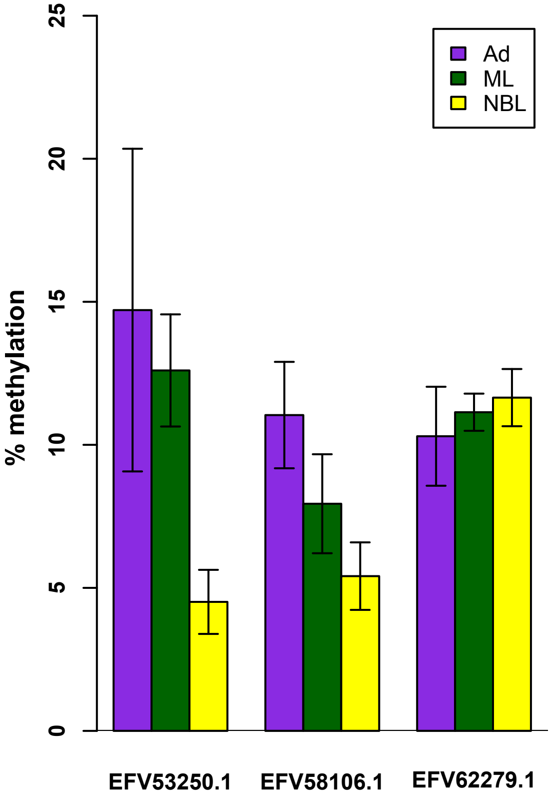

Figure S4. Results of MeDIP-QPCR validation on three randomly selected genomic regions in three life stages. The relative methylation levels of particular genomic locus among samples were compared by measuring the amount of immunoprecipitated DNA after normalization to the 10% of input DNA: %(MeDNA-IP / Total input)=2^[Ct(10%input) – 3.32 – Ct(MeDNA-IP)]×100%.

Figure S5

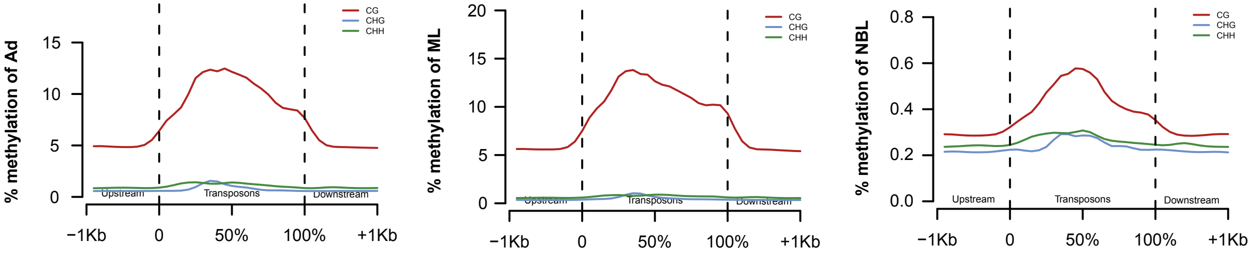

Figure S5. Distribution of methylation along TEs. 1kb upstream or downstream regions from TEs are indicated. Two vertical dashed lines mark the TE boundaries.

Figure S6

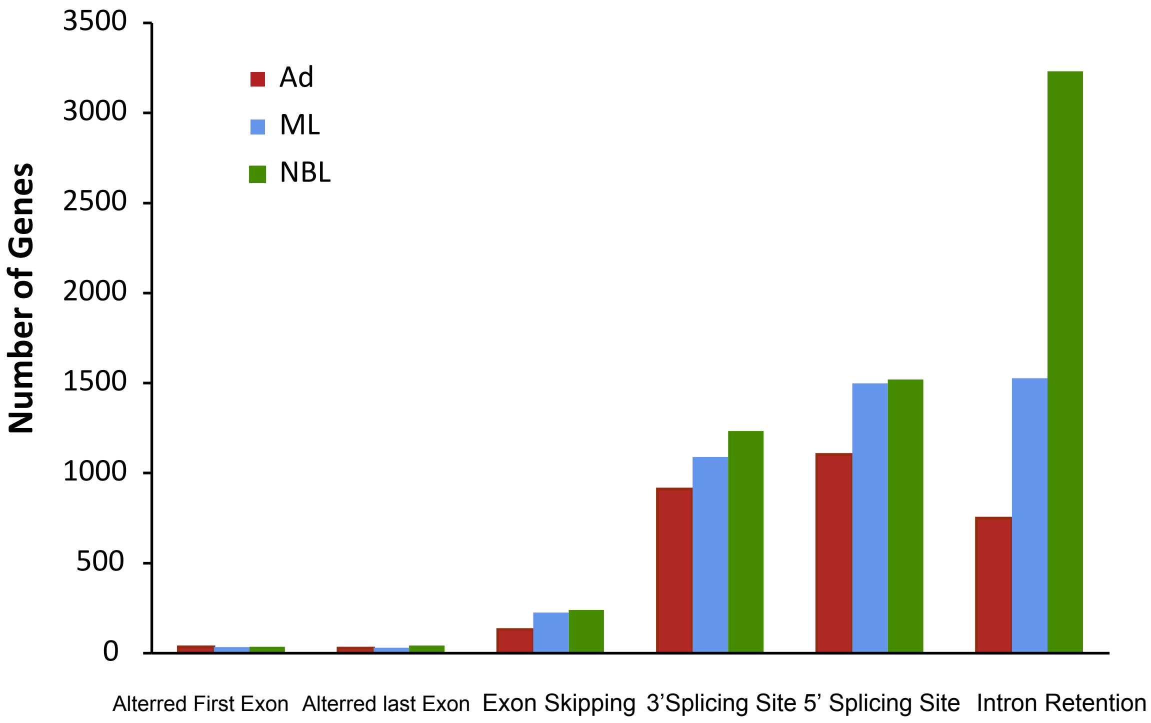

Figure S6. Summary of alternatively spliced genes in three life stages of *T. spiralis*
